# The spread of herds and horses into the Altai: How livestock and dairying drove social complexity in Mongolia

**DOI:** 10.1371/journal.pone.0265775

**Published:** 2022-05-11

**Authors:** Alicia R. Ventresca Miller, Shevan Wilkin, Jessica Hendy, Tsagaan Turbat, Dunburee Batsukh, Noost Bayarkhuu, Pierre-Henri Giscard, Jan Bemmann, Jamsranjav Bayarsaikhan, Bryan K. Miller, Julia Clark, Patrick Roberts, Nicole Boivin

**Affiliations:** 1 Department of Anthropology, University of Michigan, Ann Arbor, Michigan, United States of America; 2 Museum of Anthropological Archaeology, University of Michigan, Ann Arbor, Michigan, United States of America; 3 Department of Archaeology, Max Planck Institute for the Science of Human History, Jena, Germany; 4 Institute of Evolutionary Medicine, Faculty of Medicine, University of Zürich, Zürich, Switzerland; 5 BioArCh, Department of Archaeology, University of York, York, United Kingdom; 6 Department of Archaeogenetics, Max Planck Institute for the Science of Human History, Jena, Germany; 7 Department of Anthropology and Archaeology, National University of Mongolia, Ulaanbaatar, Mongolia; 8 Archaeological Research Center of the National University of Mongolia, Ulaanbaatar, Mongolia; 9 Institute of Archaeology, Mongolian Academy of Sciences, Ulaanbaatar, Mongolia; 10 Archaeometry Laboratory, University of Science and Technology of China, Hefei, Anhui, China; 11 Institute of Deserts and Steppes, Paris, France; 12 Prehistoric and Early Historic Archaeology, Rheinische Friedrich-Wilhelms-University of Bonn, Bonn, Germany; 13 National Museum of Mongolia, Ulaanbaatar, Mongolia; 14 History of Art Department, University of Michigan, Ann Arbor, Michigan, United States of America; 15 NOMAD Science, Glen, Montana, United States of America; 16 Department of Archaeology, Flinders University, Adelaide, South Australia, Australia; 17 Department of Sociology, Social Work and Anthropology, Utah State University, Logan, Utah, United States of America; 18 School of Social Science, University of Queensland, Brisbane, Australia; 19 Department of Archaeology, University of Calgary, Calgary, Canada; 20 Smithsonian Institution, New York, New York, United States of America; University at Buffalo - The State University of New York, UNITED STATES

## Abstract

The initial movement of herders and livestock into the eastern steppe is of great interest, as this region has long been home to pastoralist groups. Due to a paucity of faunal remains, however, it has been difficult to discern the timing of the adoption of domesticated ruminants and horses into the region, though recent research on ancient dairying has started to shed new light on this history. Here we present proteomic evidence for shifts in dairy consumption in the Altai Mountains, drawing on evidence from sites dating from the Early Bronze to the Late Iron Age. We compare these finds with evidence for the rise of social complexity in western Mongolia, as reflected in material remains signaling population growth, the establishment of structured cemeteries, and the erection of large monuments. Our results suggest that the subsistence basis for the development of complex societies began at the dawn of the Bronze Age, with the adoption of ruminant livestock. Investments in pastoralism intensified over time, enabling a food production system that sustained growing populations. While pronounced social changes and monumental constructions occurred in tandem with the first evidence for horse dairying, ~1350 cal BCE, these shifts were fueled by a long-term economic dependence on ruminant livestock. Therefore, the spread into the Mongolian Altai of herds, and then horses, resulted in immediate dietary changes, with subsequent social and demographic transformations occurring later.

## Introduction

Located in the heart of Asia, the Altai Mountains form a frontier between the western and eastern steppe, dividing distinct ecologies and populations. The ecology of this mountainous zone separates the lowlands of Kazakhstan and western Siberia, where climates are continental, from the higher elevation, and colder and drier climate, found in Mongolia [1]. Neither restrictive barrier nor freely open corridor, the Altai has instead long acted as a selectively permeable membrane enabling certain types of intersections and connections, but reducing opportunities for others. Prior to ~3500 BCE, hunter-gatherer-fisher (HGF) groups were separated, both geographically and genetically, by the Altai Mountains [2], whereas in the Bronze Age (c. 3500 to 900 BCE), populations moved into and across the mountain range in multiple waves, from regions to the west and southwest [2–4]. Recent studies on the arrival of human population waves, and transmission of cultivated species, are beginning to shed new light on the Holocene prehistory of the Altai [2, 5–14], however more work is needed to understand this region as a transmitter of economic and cultural adaptations.

The spread of farming is globally implicated in radical economic changes to societies, as well as key social, demographic and technological developments. However, in many regions of the world, the initial adoption of domesticates did not immediately lead to dramatic changes, with slower transformations instead unfolding on centennial or longer timescales [15–18]. Studies of the rise of social complexity tend to focus on shifts from HGF economies to crop cultivation, which does not fit for northern Asia, where pastoral lifeways provided a long and resilient adaptation prior to the arrival of domesticated grains [10, 19–24]. Our current data suggests that the consumption of domesticated grains did not begin in northern Mongolia until the early Iron Age (~800 BCE) [6], with no clear evidence for domesticates in the eastern Altai until the Late Iron Age (~200 BCE). One key challenge in understanding the unique pathway to social complexity in the Altai is the imprecision attached to the timing of the adoption of domesticated livestock in the region, as well as their role within changing economic systems. Faunal remains are fragmentary or comingled with contemporaneous wild counterparts, and a lack of comparative faunal collections make it difficult to differentiate the domestication status of key species [22, 25–27].

**Fig 1:**
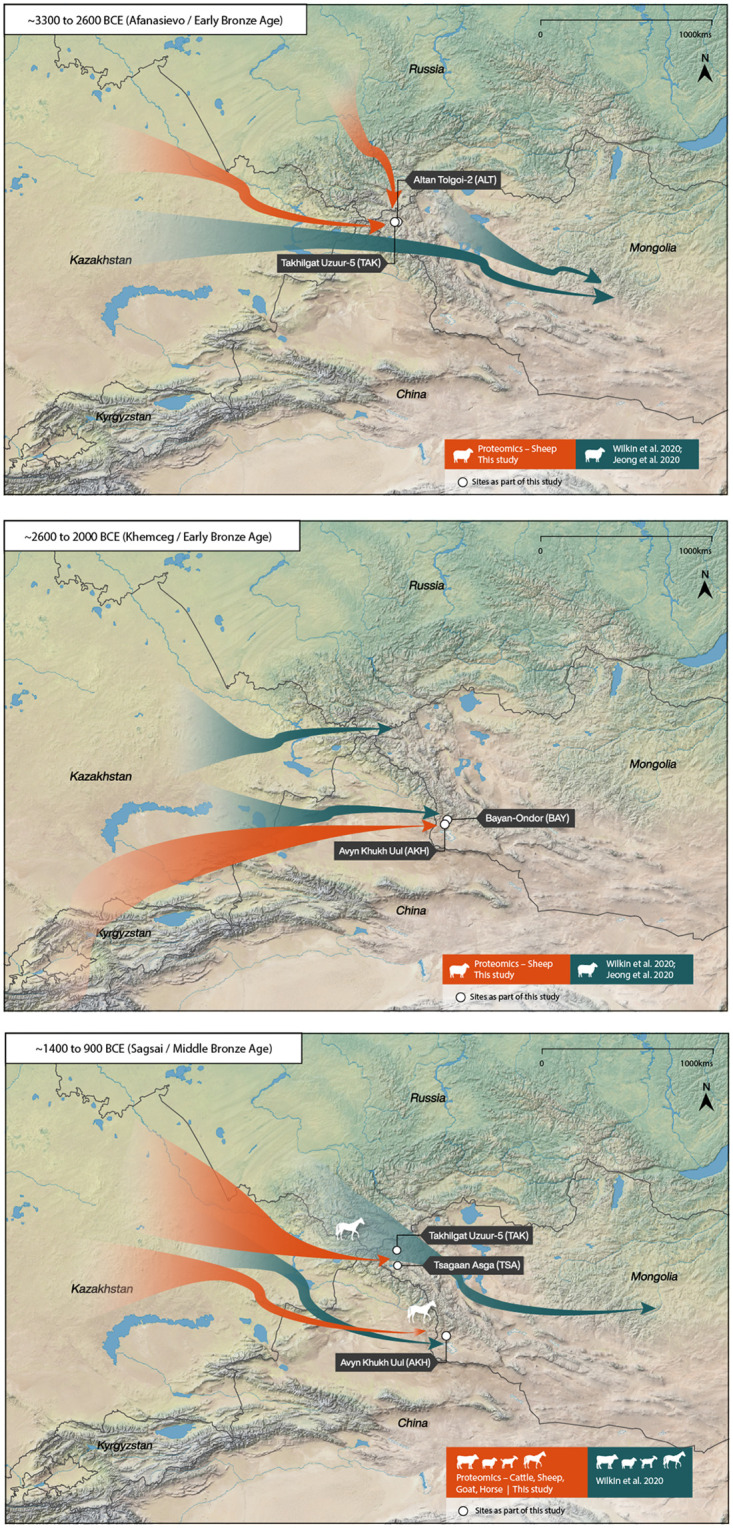
The postulated spread of herders and dairying into the Altai during the Early and Middle Bronze Ages. These maps were produced using Adobe Illustrator CC 2020 and using the Natural Early Data maps from https://www.naturalearthdta.com/downloads/ by AVM and Michelle O’Reilly (Graphic Designer for the MPI-SHH).

The application of biomolecular approaches that shed light on livestock use and dairying have begun to provide new insights into the adoption of domesticates in ancient northern Asia [5, 7–9, 14]. Proteomic research has pushed back the earliest dates for the introduction of domesticated livestock in Mongolia through the identification of milk peptides in ancient dental calculus [7]; often in periods when there are few faunal remains recovered, or those identified are indeterminate at the species level. Previous proteomic results demonstrate that in the Altai Mountains ruminant livestock were introduced by 3000 BCE, with the possibility that sheep and goats arrived before cattle [7]. As it currently stands, domesticated horses do not appear to have arrived in the region until ~1200 BCE [7]. Links between the timing of the introduction of livestock and the rise of social complexity are imprecise. However, pastoralism is an integral part of economies in the eastern Altai, a dry zone where crop cultivation is challenging and modern communities rely on ruminant livestock as their primary form of subsistence. In order to better understand the adoption of domesticated livestock in the Altai, we conducted proteomic analysis of dental calculus from 21 Bronze Age (Afanasievo, Khemceg [Chemurchek], Sagsai) and Iron Age (Xiongnu) individuals (c. 2900 BCE to 300 CE) from the region. Results of this analysis confirm that the Altai Mountains acted as a navigable barrier across which new adaptations were transmitted to the eastern steppe (Fig 1). Correlated with previous archaeological and biomolecular datasets, our results shed new light on how societies expanded and transformed after the adoption of domesticates.

## Materials and methods

### Sampling

Dental calculus samples (n = 24; individuals n = 21) were collected from ancient human populations in the Mongolian Altai dating to the Bronze and Iron Ages (Fig 1; Table 1). All samples were collected from, and analyzed with permission of, the Institute of Archaeology, Mongolian Academy of Sciences or the National Museum of Mongolia. Dental calculus was removed from permanent dentition using a sterile dental scaler. All samples were collected and stored in sterile 1.5 mL Eppendorf tubes for transfer to the Max Planck Institute for the Science of Human History Palaeoproteomics Laboratory.

**Table 1:** Total dairy per individual.

Lab Number	Original Sample Number	Culture	Calibrated (OxCal)	Protein	Total Dairy Peptides	Dietary Proteins	OSSD Score
**Z690**	**DA-ALT-002**	**Afanasievo (EBA)**	**Individual associated with DA-ALT-04**	**BLG**	**23**	***Ovis*, Bovidae, *Ovis*/Bovinae**	**82**
**Z691**	**DA-ALT-004**	**Afanasievo (EBA)**	**2912–2671 cal BC 95.4 (2 sigma)**	**BLG**	**39**	***Ovis*, Caprinae, *Ovis*/Bovinae, Bovidae**	**75**
**Z687**	**DA-TAK-005**	**(EBA)**	**2873–2580 cal BC 95.4 (2 sigma)**	**BLG**	**48**	***Ovis*, *Ovis*/Bovinae, Caprinae, Bovidae**	**68**
				**Alpha S1 casein**	**4**	**Caprinae, Bovidae**	
				**Alpha-lactalbumin**	**2**	**Caprinae**	
				**Putative germin-like protein**	**2**	**Poaceae**	
				**Germin-like protein 8–10**	**2**	**Poaceae**	
**Z696**	**DA-AKH-020**	**Khemceg / Chemurchek (EBA)**	**2623-2457cal BC 95.4 (2 sigma)**	**BLG**	**39**	***Ovis*, Bovidae**	**55**
				**Alpha S1 casein**	**6**	**Bovidae, Caprinae**	
**DA513**	**DA-BAY-001**	**Khemceg / Chemurchek (EBA)**	**2623–2464 cal BCE 95.4 (2 sigma)**	**−**	**0**	**No Dietary Proteins**	**36** *
**Z692**	**DA-BAY-001**	**Khemceg / Chemurchek (EBA)**	**2623–2464 cal BCE 95.4 (2 sigma)**	**−**	**−**	**FAILED EXTRACTION**	**N/A** *
**Z682**	**DA-TSA-10**	**Sagsai (MBA)**	**1504–1328 cal BC 95.4 (2 sigma)**	**−**	**0**	**No Dietary Proteins**	**57** *
**DA380**	**DA-TSA-10**	**Sagsai (MBA)**	**1504–1328 cal BC 95.4 (2 sigma)**	**BLG**	**8**	***Ovis*, *Ovis*/Bovinae**	**75** *
**Z685**	**DA-TSA-058**	**Sagsai (MBA)**	**1424–1270 cal BC 95.4 (2 sigma)**	**BLG**	**8**	***Ovis*, *Ovis*/Bovinae, Caprinae**	**50**
				**BLG I**	**4**	**Equus**	
**Z689**	**DA-TAK-021**	**Sagsai (MBA)**	**1428–1235 cal BC 95.4 (2 sigma)**	**BLG**	**3**	**Pecora**	**45**
**Z686**	**DA-TAK-001**	**Sagsai (MBA)**	**1427–1234 cal BC 95.4 (2 sigma)**	**BLG**	**92**	**Capra hircus, Bovidae, Caprinae, Ovis/Bovinae, Bovinae**	**86**
				**Alpha S1 casein**	**7**	**Caprinae, Bovidae**	
				**Alpha-lactalbumin**	**4**	**Caprinae**	
				**Beta-casein**	**2**	**Bovidae**	
**Z683**	**DA-TSA-033**	**Sagsai (MBA)**	**1399–1134 cal BCE 95.4 (2 sigma)**	**−**	**−**	**FAILED EXTRACTION**	**N/A** *
**DA379**	**DA-TSA-033**	**Sagsai (MBA)**	**1399–1134 cal BCE 95.4 (2 sigma)**	**BLG**	**21**	**Ovis, Ovis/Bovinae**	**63** *
				**BLG 1**	**12**	***Equus***	
				**Lysozyme C, milk isozyme**	**7**	***Equus***	
**Z694**	**DA-AKH-001**	**Sagsai (MBA)**	**1259–1016 cal BCE 95.4 (2 sigma)**	**BLG**	**35**	***Capra hircus*, *Ovis*/Bovinae, Caprinae**	**59**
				**Alpha S1 casein**	**3**	**Caprinae, Bovidae**	
				**BLG 1**	**2**	***Equus***	
**Z688**	**DA-TAK-013**	**Sagsai (MBA)**	**1366–1050 cal BCE 95.4 (2 sigma)**	**BLG**	**44**	***Capra hircus*, Bovidae, *Ovis*, *Ovis*/Bovinae, Caprinae**	**82**
				**Alpha S1 casein**	**2**	**Caprinae, Bovidae**	
				**BLG**	**3**	***Equus***	
**Z693**	**DA-KHO-001**	**Sagsai (MBA)**	**1218–1002 cal BCE 95.4 (2 sigma)**	**−**	**−**	**FAILED EXTRACTION**	**N/A**
**Z684**	**DA-TSA-051**	**Sagsai (MBA)**	**1214–1015 cal BCE 95.4 (2 sigma)**	**−**	**−**	**FAILED EXTRACTION**	**N/A**
**Z695**	**DA-AKH-016**	**Xiongnu (LIA)**	**46 cal BCE—121 cal CE 95.4 (2 sigma)**	**No Dairy**	**0**	**No Dietary Proteins**	**35**
**Z697**	**DA-SBR-001**	**Xiongnu (LIA)**	**−**	**BLG I**	**2**	***Equus***	**50**
**Z698**	**DA-SBR-004**	**Xiongnu (LIA)**	**−**	**No Dairy**	**0**	**FAILED EXTRACTION**	**N/A**
**Z699**	**DA-SBR-007**	**Xiongnu (LIA)**	**−**	**No Dairy**	**0**	**FAILED EXTRACTION**	**N/A**
**Z700**	**DA-SBR-014**	**Xiongnu (LIA)**	**129–243 cal CE 95.4 (2 sigma)**	**No Dairy**	**0**	**No Dietary Proteins**	**58**
**Z701**	**DA-SBR-016**	**Xiongnu (LIA)**	**−**	**No Dairy**	**0**	**No Dietary Proteins**	**33**
**Z702**	**DA-SBR-017**	**Xiongnu (LIA)**	**−**	**No Dairy**	**0**	**FAILED EXTRACTION**	**N/A**

*denotes proteins were extracted twice using different methods.

### Archaeological sites sampled

Samples were collected from seven archaeological sites located in the Altai Mountains of western Mongolia. The sites range in date from the Early Bronze through Late Iron Ages (~2900 cal BCE to 240 CE) (Table 1). The earliest individuals in the sample set are from the site of Altan Tolgoi 2 (ALT-02/ALT-04); they were buried in a single grave and date to the Early Bronze Age (2912–2671 cal BCE). Located nearby was the site of Takhilgat Uzuur 5, which included burials of individuals from multiple time periods. The earliest burial at the site (TAK-05) included a single individual associated with the Early Bronze Age (EBA) Afanasievo culture (2873–2580 cal BCE). Three other burials at Takhilgat Uzuur 5 (TAK-21, TAK-01, and TAK-13) included individuals from the Middle Bronze Age (MBA) Sagsai culture, dating to 1428–1235 cal BCE, 1427–1234 cal BCE, and 1366–1050 cal BCE respectively.

The site of Avyn Khukh Uul included burials that spanned three different periods of time. One burial (AKH-20) was associated with the Khemceg culture (EBA), with a date from 2623–2457 cal BCE. A second individual from this site (AKH-01) dated to the Middle Bronze Age (1259–1016 cal BCE). Finally, another burial (AKH-16) contained an individual dating to the Late Iron Age (LIA) Xiongnu era (46 cal BCE—121 cal CE). A further individual associated with the EBA Khemceg culture was identified at the site of Bayan Ondor; dental calculus of a single individual was examined, with a radiocarbon date of 2623–2464 cal BCE.

The site of Tsagaan Asga was comprised of burials from a single period, the Middle Bronze Age, associated with the Sagsai culture. We analyzed four individuals (TSA-10, TSA-58, TSA-33, and TSA-51) from the site, with radiocarbon dates spanning from 1504 to 1015 cal BCE (S2 Table in S1 File). Dental calculus from a single individual from the site of Khokh Uzuur-1 (KHO-01), dated to the Middle Bronze Age (1218–1002 cal BCE), was also analyzed.

The site of Shombuuzin-Belchir includes burials that date to the Late Iron Age (LIA) or Xiongnu Empire period. We analyzed individuals from six tombs (SBR-01, SBR-04, SBR-07, SBR-14, SBR-16, and SBR-17). Only a single individual from one tomb revealed evidence of dietary proteins (SBR-01), and only one of the individuals from the site has been radiocarbon dated (SBR-14) with a range of dates from 129–243 cal CE.

### Protein extraction

Proteins were extracted from each sample in a dedicated paleoproteomics lab in the Department of Archaeology at the Max Planck Institute for the Science of Human History. All dental calculus samples were demineralized with 0.5 M EDTA (pH 8.0), followed by further steps for denaturation, reduction and alkylation. Proteins were digested with trypsin overnight, and peptide clean-up was completed on C18 StageTips. For all samples with a “Z” designation, a Filter Aided Sample Preparation (FASP) protocol was used, as described in a previous publication [5]. Samples with a “DA” designation were extracted with a modified single pot, solid phase, extraction protocol (SP3) modified for use on human dental calculus [14, 28]. Three samples, (TSA-10, TSA-33, and BAY-01) were extracted twice, once with a FASP protocol and again with an SP3 protocol. The full FASP protocol was published previously [7] and the full SP3 protocol is available at protocots.io (dx.doi.org/10.17504/protocols.io.bfgrjjv6) [14]. The resulting digested peptides from both extraction methods were stored at -20°C before shipping to the Functional Genomics Center Zürich, at the University of Zürich in Zürich, Switzerland for analysis by liquid chromatography, tandem mass spectrometry (LC-MS/MS).

### LC-MS/MS analysis

Mass spectrometry (LC-MS/MS) was conducted at the Functional Genomics Center Zurich using either a Q-Exactive or a Q-Exactive HF mass spectrometer (Thermo Scientific, Bremen, Germany) equipped with a Digital PicoView source (New Objective) and coupled to a nanoACQUITY or an ACQUITY UPLC M-Class system (Waters AG, Baden-Dättwil, Switzerland), respectively. Solvent composition at the two channels was 0.1% formic acid for channel A and 0.1% formic acid, 99.9% acetonitrile for channel B. Column temperature was 50°C. For each sample 4 μL of peptides were loaded on a commercial MZ Symmetry C18 Trap Column (100Å, 5 μm, 180 μm x 20 mm, Waters) followed by nanoEase MZ C18 HSS T3 Column (100Å, 1.8 μm, 75 μm x 250 mm, Waters). The peptides were eluted at a flow rate of 300 nL/min by a gradient from 8 to 22% B in 49 min, 32% B in 11 min and 95% B in 1 min (Q-Exactive) or from 5 to 40% B in 120 min and 98% B in 5 min (Q-Exactive HF). The column was cleaned after each run with 98% solvent B for 5 min and holding 98% B for 8 min prior to re-establishing loading condition.

The mass spectrometers were operated in data-dependent mode performing HCD (higher-energy collision dissociation) fragmentation on the twelve most intense signals per cycle. The settings were slightly adapted for each instrument. For Q-Exactive analyses, full-scan MS spectra (300–1700 m/z) were acquired at a resolution of 70,000 at 200 m/z after accumulation to a target value (AGC) of 3E6, while HCD spectra were acquired at a resolution of 35,000 using a normalized collision energy of 25 (maximum injection time: 110 ms; AGC 50,000 ions). For Q-Exactive HF analyses, full-scan MS spectra (300–1500 m/z) were acquired at a resolution of 120,000 at 200 m/z after accumulation to a target value (AGC) of 3,000,000, while HCD spectra were acquired at a resolution of 30,000 using a normalized collision energy of 28 (maximum injection time: 50 ms; AGC 10,000 ions). Unassigned singly charged ions and ions were excluded. Precursor masses previously selected for MS/MS measurement were excluded from further selection for 30 s, and the exclusion window was set at 10 ppm. The samples were acquired using internal lock mass calibration on m/z 371.1012 and 445.1200.

### Data analysis

We converted raw LC-MS/MS spectra to Mascot generic files (MGF) using MSConvert from ProteoWizard (v.3.0.11781) by selecting the top 100 peaks. MS/MS database searches were performed using Mascot (v.2.6.0) against available sequences in a custom curated Dairy Database (Wilkin et al. 2020a) and SwissProt, with both databases decoyed with a full reverse listing of all sequences in order to calculate the false discovery rate. Mascot settings included the instrument type set to Q-Exactive and the enzyme set to trypsin. Peptide mass tolerance was set at 10 ppm, with fragment mass tolerance of 0.01 Da, with an allowance for monoisotopic mass shifts. As we reduced and alkylated the disulfide bonds, carbamidomethyl of cystine (C) was set as a fixed modification, and based on previous observations of modifications detection in ancient dental calculus [29–31], we chose deamidation of asparagine (N) and glutamine (Q) and oxidation of methionine (M) as our variable modifications.

Peptide search results were additionally filtered using the custom R script MS-MARGE (E-value of between 0.01 or 0.001, depending on the resulting false discovery rate (FDR)) [32]. MS-MARGE calculates the FDR by comparing the number of forward sequence hits to the number of reverse sequence hits. This allows the user to adjust the E-value cutoff manually in order achieve an appropriate FDR for each sample. After data filtration, our average peptide FDR was 0.84 with an average protein FDR of 1.93. Peptide homology was additionally tested using BLASTp. All raw, processed and result files are available via the ProteomeXchange under accession PXD029267.

We further conducted an analysis of the overall proteome preservation of each dental calculus sample [28]. Using a curated Oral Signature Screening Database (OSSD), we looked for a combination of lab contaminants, common contaminants introduced through contact with archaeological and curation contexts, common bacteria found in the human microbiome, and human immune proteins that are regularly identified in the oral cavity. While Bleasdale and colleagues [28] applied the OSSD database to their raw MS/MS data as a screening device, we searched our filtered results against the proteins included in Bleasdale’s OSSD as a way to test the relative preservation of all the samples in our study. We compared the number of proteins commonly identified within the oral cavity (oral microbiome and salivary immune proteins) against a total count of proteins identified from the four categories combined. Ancient protein studies on human dental calculus [28, 33, 34] have demonstrated that the overall proteome for archaeological samples generally consists of proteins from four major sources: those produced during immune response found within saliva, oral microbiome bacteria, environmental contaminants (from the soil environment, human handling in excavation and curation, and cross contamination from storage or display near other human and animal skeletons), and laboratory contaminants (introduced during extraction protocols and commonly found in mass spectrometry facilities and reagents). Here we quantify the overall oral signature preservation by comparing the number of immune oral bacteria proteins identified over the total proteins identified in the sample. For this study, an OSSD score of 45 is considered passing. Thus, at least 45% (of the total count) of proteins were associated with common oral microbiome or salivary immune proteins. The overall proteome can shift depending on diet, region, and environment of the site under study. A cut off of 45 was decided after scoring all samples and evaluating each recovered proteome. Samples with a score under 45 had a far higher number of contamination peptides and had a proteome that contained more proteins associated with contaminates than expected, for a sample to be considered to represent an authentic oral signature.

## Results

Of the 24 dental calculus samples (from 21 individuals) analyzed, 71% resulted in successful protein extractions (17 of 24) while 29% resulted in failed extractions (S1 Table in S1 File). Successful extractions were subjected to an OSSD screening, to quantify evidence of an ancient dental calculus oral signature, which typically includes human salivary and immune proteins, as well as those from bacteria common in the human oral microbiome [33, 34] (S1 Fig in S1 File). While 17 extractions were successful, only 14 samples (82%) had OSSD scores of 45 or greater. Of the 14 samples that passed screening, 12 showed evidence for consumption of dairy, while another 2 revealed no dietary proteins (S1 Table in S1 File).

### Identified proteins

**Fig 2:**
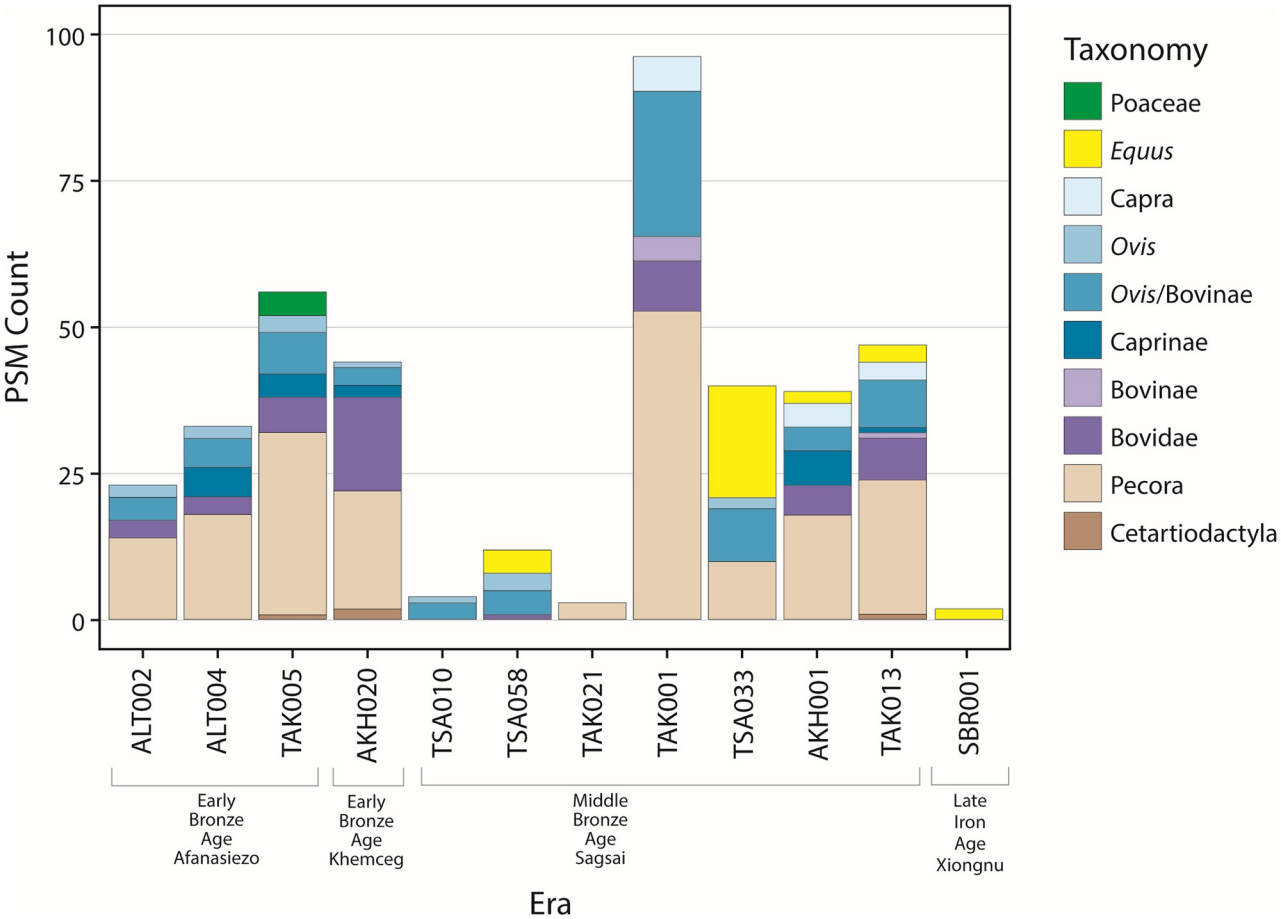
Bar plot showing the peptide spectral matches (PSMs) for each dietary protein per individual. Note the lack of dietary proteins in the SBR individuals, and see Supporting Note 1 and S1 Fig in S1 File. for corresponding preservation information for each individual.

In the samples (n = 14) that produced a typical oral protein signature and passed OSSD screening, dairy proteins were the primary dietary proteins identified, in line with previous observations of dietary proteins in ancient dental calculus [28, 34, 35]. These identified proteins included the whey protein β-lactoglobulin (BLG), which was present in 8 samples; β-casein, identified in 1 sample; alpha-lactalbumin, identified in 2 samples; and α-S1-casein, identified in 3 samples. β-lactoglobulin I was identified, as well as the milk isozyme lysozyme C, in samples with horse milk proteins (Fig 2). In one Early Bronze Age (EBA) individual (TAK-05), evidence for two plant proteins specific to the Poaceae family were also detected.

### EBA dairy results (Afanasievo and Khemceg [Chemurchek] cultures)

The earliest individuals (n = 5) studied here consisted of 6 calculus samples dating to the Early Bronze Age (2912–2457 cal BC). Four successfully extracted samples contained peptide evidence for ruminant dairy consumption, most commonly in the form of BLG, but also alpha-S1-casein and alpha lactalbumin (Table 1). Most of the identified dairy peptides were specific to the Pecora infraorder (cattle, yak, sheep, goat, water buffalo, reindeer) or Bovidae family (cattle, sheep, goat), with others specifically indicative of caprines (sheep, goat). Additionally, all four samples included species-specific peptides for *Ovis* (sheep) as well as those of animals of higher taxonomic classification. Only one individual (BAY-01) had dental calculus that resulted in a failed extraction (Z692) using the FASP protocol, while a second extraction (DA513) with the SP3 protocol was successful, but did not contain any dietary proteins. The single EBA calculus sample without evidence for dairy peptides (DA513/BAY-01) suffered from extremely poor preservation of the ancient oral proteome. In contrast to what was observed in well-preserved samples, contaminant proteins in this sample were dominant (S1 Fig in S1 File); with very few oral immune proteins present, this sample did not pass our oral signature protein preservation score threshold (S1 Table in S1 File).

### MBA dairy results (Sagsai culture)

Dental calculus from 9 individuals (11 calculus samples) dating to the Middle and Late Bronze Ages (1504–1015 cal BCE) were studied. Of these, 3 were failed extractions (Z683, Z693, Z684) and one successful extraction lacked dietary proteins (Z682). The individual that did not have evidence for dairy consumption (Z682/TSA-10) also had little evidence for an oral signature normally found in well-preserved calculus samples (S1 Fig, S1 Table in S1 File). The 7 remaining samples were well-preserved and contained evidence for ruminant dairy, with 5 individuals containing the milk of at least two distinct species. Of these individuals, 4 had calculus with evidence for *Ovis*-specific BLG peptides, 3 contained peptides specific to *Capra hircus*, and all contained peptides with less specific taxonomic classifications (Caprinae, Bovidae, Pecora) (Table 1). Evidence for horse milk consumption was present in 3 individuals in the form of BLG I and Lysozyme C (milk isozyme)—all peptides specific to *Equus*. The earliest individual showing evidence for horse milk consumption was dated to c. 1350 BCE (1424–1270 cal. BCE), which is 150 years earlier than previously published results for *Equus* milk on the eastern steppe [7] and ~100 years earlier than published radiocarbon results from Equus osteological remains [36].

### Late Iron Age dairy results (Xiongnu culture)

The Late Iron Age sites in the Altai featured 7 individuals, with only 2 samples successfully extracted. These sites date to the Xiongnu era, spanning from 46 cal BC to 243 cal CE. Of the 2 individuals that contained abundant proteins associated with an oral signature and passed the OSSD preservation screening, one lacked dietary proteins (Z700/SBR-14) while the other individual (Z697/SBR-01) had dental calculus containing *Equus*-specific peptides from BLG I.

## Discussion and conclusion

Our results provide supporting proteomic evidence for extensive, multi-species dairying in the Altai from at least the Early Bronze Age. Together with previously reported data from the region [2, 5, 7], they also point to widespread consumption of milk amongst Altai populations throughout the later Bronze Age. Detection of taxa-specific dairy peptides demonstrate that sheep, goat, cattle and horse were all being milked, as was also the case during the Bronze Age in the Pontic-Caspian region far to the west [14]. The identification of the earliest horse milk peptides in the region pushes back the evidence for horse milk drinking amongst Altai populations to c. 1350 BCE. When analyzed in tandem with evidence from well-documented archaeological sites, as well as recent ancient DNA results [2–5, 9], these proteomic findings provide useful new data for assessing broader dietary and economic changes. Here we bring the archaeological and paleoproteomic evidence together to explore the broader implications of the emerging dairying evidence.

### Transitioning to pastoral lifeways

The archaeological record of pre-pastoral hunter-gatherer-fisher (HGF) groups in the Mongolian Altai before ~3500 BCE is reflected in surface scatters of lithics and ceramics, which provide evidence for residential areas [22], but no pithouses or other structures. Beginning around 3000 BCE, or earlier, the first herders and their ruminants crossed geographic and cultural boundaries, bringing knowledge of livestock management and milking into Mongolia [7]. Our results provide direct evidence (2912–2761 cal BC) for consumption of sheep (*Ovis*) and other unspecified ruminant (Bovidae, Caprinae) milk among Afanasievo groups in the Altai during the Early Bronze Age (EBA). Burials were, for the first time, marked by large stone circles (up to 15 meters in diameter) or rectangles (~7x10 meters) [37]. Often found as single burials, Afanasievo (EBA) graves include ruminant remains and early metal artifacts [38]. Within the burials excavated in the Altai, sheep and goat remains were recovered (Supporting Note 1 in S1 File). Ancient DNA results indicate that individuals in the region at this time had genetic profiles similar to individuals from the Yenisei region, located north of the Altai range [2–4]. This new demarcation of graves, the presence of ruminants, and the genetic ancestry of the individuals buried in Afanasievo graves likely point to the influx of a new culture. Current data suggests that the Afanasievo were coming from the western steppe or regions to the north [2, 9]. While EBA Afanasievo herders were conceivably the first to bring dairy pastoralism to Mongolia, this group did not leave a long-lasting genetic presence in the region [2] nor any evidence indicative of the complex social structures and population growth later observed in the region.

**Fig 3:**
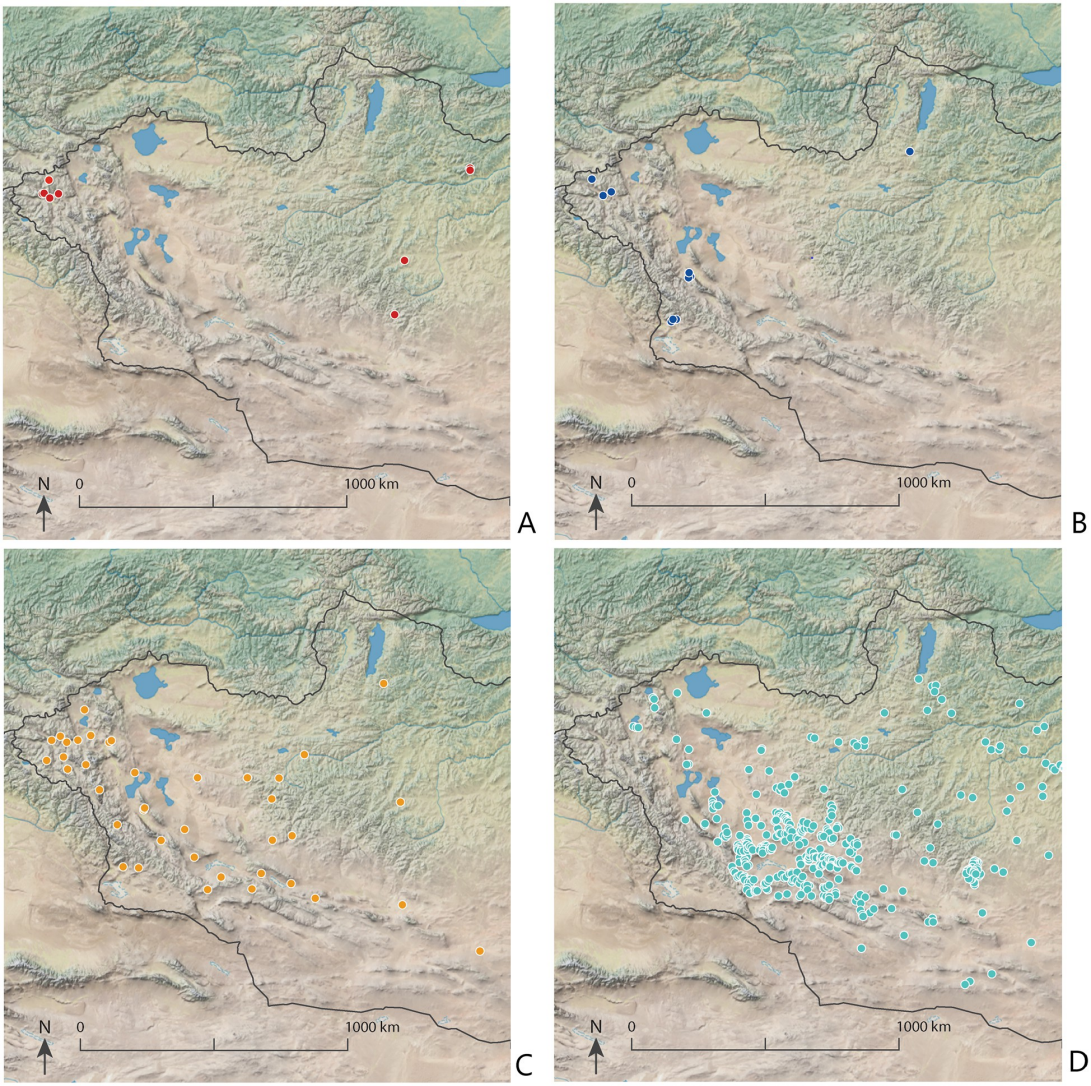
Archaeological sites identified in the Altai from the Early to Middle/Late Bronze Age point to a demographic increase beginning in the Middle Bronze Age (partially based on maps in [42]). These maps were produced using Adobe Illustrator CC 2020 and using the Natural Early Data maps from https://www.naturalearthdta.com/downloads/ by AVM and Michelle O’Reilly (Graphic Designer for the MPI-SHH) and John Klausmeyer (Illustrator for the UMMAA). A) Early Bronze Age (Afanasievo); B) Early Bronze Age (Khemceg or Monkhkhairkhan); C) Middle Bronze Age (Sagsai); D) Middle/Late Bronze Age (Khirgisuur).

Subsequently, new populations moved into and across the Altai in at least two waves, from the west and then, later, from the southwest (Fig 1) [2]. Our results indicate that as early as 2500 BCE, Early Bronze Age Khemceg (Chemurchek) individuals were consuming sheep (*Ovis*) and ruminant (Caprinae) milk. Buried alongside the individuals we analyzed from this period were the osteological remains of sheep, goat, and cattle (Supporting Note 1 in S1 File). The size of burial markers increases in this period, with circular stone mounds reaching 20 meters in diameter [39]. The Khemceg culture (2700–1900 BCE) is characterized by individual burials with sizeable anthropomorphic standing stones and motifs linked to cultures found to the north [39]. In the Altai, the Khemceg culture shares a genetic affinity with HGF groups (Ancient North Eurasian) as well as populations to the southwest (Bactria-Margiana) [2]. We postulate that these later populations first introduced domesticated cattle to Mongolia for dairying, based on osteological remains (Supporting Note 1 in S1 File). A new culture and economy, as well as ties to larger social networks, may be linked to an initial burst of social complexity, evidenced in greater investment in mortuary rituals (see Fig 3).

### Early horse dairying and population growth

During the Middle Bronze Age, the Altai again saw an influx of new populations, this time with genetic ties to Sintashta culture groups far to the west [2, 40]. Among these populations in the Altai, referred to as the Sagsai culture, we identified early evidence for horse milk consumption (*c*. 1350 BCE) and our findings confirmed the consumption of sheep (*Ovis)* and goat (*Capra hircus)* dairy products. The earliest evidence for the Sagsai culture 1500–980 BCE [21] occurred in the Altai Mountains, with later evidence in northern and central Mongolia. The Sagsai culture was the first to build enclosures with standing stones placed around stone mounds [21, 41]. This is also the first time that planned cemeteries, as opposed to burials arranged on their own or in very small clusters, have been identified in the region [21]. Within Sagsai cemeteries that are well-dated, burial traditions include both round and square tombs of varying sizes (Supporting Note 1 in S1 File). The proliferation of archaeological sites across Mongolia during the Middle Bronze Age suggests that populations continued to grow and expand into new regions of the steppe [24, 42] (Fig 3). Population growth is further supported by systematic survey in the Middle Gobi region of Mongolia [43] (Supporting Note 2 in S1 File).

Sagsai burials (1500–970 cal BCE) were present slightly earlier than, but also contemporaneous with, elaborate stone monuments called khirgisuurs, which date from 1200–750 BCE [44]. Sagsai burial monuments have large mounds with enclosures, similar to the construction of Khirgisuurs [21, 24, 41]. At sites with both Sagsai burials and Khirgisuurs the former date to earlier periods [45]. The complex nature of some Khirgisuur complexes provide evidence for concentrated labor, although whether this was as part of socially integrative events [46] or reflects emerging systems of social inequality [47] remains disputed. At Khirgisuur sites across Mongolia, much attention has been paid by archaeologists to the ritual deposit of horse heads and hooves, while evidence for the sacrifice of ruminants and their placement in stone circles is often overlooked (but see [48, 49]). However, the ritual deposits of horses were a regional phenomenon and Khirgisuurs in the Altai only contain deposits of ruminants [50]. An economic reliance on ruminant livestock is supported by well-documented Bronze Age burial sites in northern Mongolia (1380 to 975 BCE), where there is a strong ruminant dairy profile in human dental calculus, but a lack of evidence for horse milk consumption [5]. A dietary focus on livestock is also well supported by isotopic research on individuals from these periods [6, 10] and archaeological research of settlements [48]. Based on accumulated proteomic and archaeological evidence, hierarchal social structures in Mongolia appeared a millennium or more after domesticated ruminants were first introduced. This gap between the initial ruminants and the intensification of ruminant production, indicates that we can identify different stages in the introduction of domesticates. As marked changes in burial styles and traditions occurred following the introduction of domesticated ruminants and dairying practices, we suggest that long-term population growth, as a result of the use of domesticated ruminants and increased regional carrying capacity, catalyzed dramatic shifts in population size and social complexity (Fig 3).

Our identification of the earliest direct evidence for horse dairy consumption among two Sagsai individuals (1427–1277 cal BCE and 1396–1155 cal BCE) highlights the importance of the Middle Bronze Age as a formative period. In comparison to the number of pre-1500 BCE monumental burials, the dramatic increase in the number and size of Sagsai burial monuments provides evidence for population expansion, cultural differentiation, and the rise of inequality during the Bronze Age. Ruminant dairy has consistently been identified among Altai populations, including the Sagsai, where ruminant peptides were identified in abundance. While the timing of early horse dairying coincides with the construction of Sagsai cemeteries, it is clear that horses were relatively novel and not an economic mainstay of these economies. Domesticated horses occupied a primary role in ritual life, yet ruminant livestock were the foundation of pastoralist subsistence economies that drove population growth and the rise of monumental architecture.

Peaks in social complexity are often driven by long-term growth in human populations that occur after subsistence diversification [51], for example the addition of ruminant animals and dairy to HGF economies. Cultural tipping points [52], or flash points, are associated with shifts in cosmology, climate, or the introduction of new technologies. In the Altai, this tipping point occurred as a result of multiple waves of human populations and accompanying ruminants moving across the steppe and Altai Mountains into new ecological zones inhabited by HGF populations. While these new pastoral foodways eventually increased economic stability, there was a maturation period between the initial adoption of domesticated livestock and the viable management of herds. The integration of herds into societies was a gradual process, during which populations grew and herds expanded. Over long time scales, advances in technologies and knowledge supported the management and survival of livestock. Population growth is tied to economic advances, which permitted an increase in the number and scale of monumental complexes. As such, the Mongolian Bronze Age stands as a formative period for the rise of social complexity in eastern Eurasia.

## Supporting information

S1 File (DOCX)
