# Supplementary material for: The spread of herds and horses into the Altai: How livestock and dairying drove social complexity in Mongolia
Source: PLoS One. 2022 May 11;17(5):e0265775. doi: 10.1371/journal.pone.0265775 (PMC9094512; doi:10.1371/journal.pone.0265775)
Supplement: S1 File — (DOCX) [file pone.0265775.s001.docx]

**SUPPORTING INFORMATION**

**The spread of herds and horses into the Altai: How livestock and dairying drove social complexity in Mongolia**

Alicia R. Ventresca Miller^1,2,3*^, Shevan Wilkin^3,4^, Jessica Hendy^5,6^, Tsagaan Turbat^7,8*^,
Dunburee Batsukh^9^, Noost Bayarkhuu^10^, Pierre-Henri Giscard^11^, Jan Bemmann^12^, Jamsranjav Bayarsaikhan^3,13^, Bryan K. Miller^2,3,14^, Julia Clark^15,16,17^, Patrick Roberts^3,18^, Nicole Boivin^3,18,19,20^

**Author Affiliations**

^1^ Department of Anthropology, University of Michigan, Ann Arbor, Michigan 48109

^2^ Museum of Anthropological Archaeology, University of Michigan, Ann Arbor, Michigan, 48109

^3^Department of Archaeology, Max Planck Institute for the Science of Human History, Kahlaische Strasse 10, 07745 Jena, Germany

^4^Institute of Evolutionary Medicine, Faculty of Medicine, University of Zürich, 8057 Zürich, Switzerland

^5^BioArCh, Department of Archaeology, University of York, York, UK, YO104DE

^6^Department of Archaeogenetics, Max Planck Institute for the Science of Human History, Kahlaische Strasse 10, 07745 Jena, Germany

^7^Department of Anthropology and Archaeology, National University of Mongolia, Ikh Surguuliin Street-1, Ulaanbaatar, Mongolia.

^8^Archaeological research center of the National University of Mongolia, Ikh Surguuliin Street-1, Ulaanbaatar, Mongolia

^9^Institute of Archaeology, Mongolian Academy of Sciences, Ulaanbaatar, Mongolia

^10^Archaeometry laboratory, University of Science and Technology of China, Hefei 230026, Anhui, China

^11^Institute of Deserts and Steppes, Paris, France

^12^Prehistoric and Early Historic Archaeology, Rheinische Friedrich-Wilhelms-University of Bonn, Germany

^13^National Museum of Mongolia, Juulchin Street-1, Ulaanbaatar, Mongolia

^14^History of Art Department, University of Michigan, Ann Arbor 48109, Michigan, United States

^15^NOMAD Science, [www.nomadsciencemongolia.com](http://www.nomadsciencemongolia.com/), Glen, Montana 59732

^16^Flinders University: Department of Archaeology, Flinders University, Bedford Park, 5042 Adelaide, SA, Australia 84322

^17^Department of Sociology, Social Work and Anthropology, Utah State University, Logan, Utah

^18^School of Social Science, University of Queensland, Brisbane, Australia

^19^Department of Archaeology, University of Calgary, Calgary, Canada

^20^Smithsonian Institution, New York, USA

**Supporting Information:**

**Supporting Note 1. Archaeological Background**

**Supporting Note 2. Demographic Change**

**Supporting Figures:**

**S1 Fig. Total proteins per sample based on OSSD results**

**S2 Fig. Spectra** (a,d) DA687 (a) Putative Germin-like protein 3-4 specific to Pocaecae, (d) Germin-like protein 8-10; (b,e) Z691 (b) final tryptic peptide from BLG, specific to Caprinae (*Ovis* or *Capra*), (e) Most commonly recovered BLG peptide, which is specific to Bovinae (cow, yak, water buffalo). Importantly the second Aspartic Acid (D) residue in the sequence has the same mass as a deamidated Aparagine (N) which would be specific to *Ovis*; (c,f) DA379 both spectra are for peptides from BLG I specific to *Equus*. Horses produce both BLG I and II proteins, which are genetically present in ruminants, only the BLG I is expressed.

**Supporting Tables:**

**S1 Table. Results of OSSD with total proteins identified, score (%), and whether the sample passed or failed the initial assessment**

**S2 Table. Radiocarbon Results**

**Supporting Note 1. Archaeological Background**

Samples of human dental calculus were collected from six archaeological sites located in the Altai Mountains of western Mongolia. The sites range in date from the Early Bronze through Late Iron Ages (~2800 cal BCE to 200 CE) (S2 Table). No permits were required for the described study, which complied with all relevant regulations. All dental calculus samples from the Bronze and Iron Ages were collected from the Institute of Archaeology, Mongolian Academy of Sciences, with the exception of the Iron Age site of Shombuuzyn-Belchir (SBR). SBR dental calculus samples were collected from the National Museum of Mongolia. These institutions are both located in Ulaanbaatar, Mongolia.

**Early Bronze Age - Afanasievo Culture**

The earliest individuals in the sample set are from the site of ***Altan Tolgoi 2*** (AT-2, Tomb 1, Individuals A and B), which were buried in a single grave that produced a conventional radiocarbon age of 4228±40 (2814-2678 cal BCE). On the surface, the tomb was marked by a rectangular stone mound measuring 6 x 9.4 meters. An outer ring of larger stones was identified along the periphery with a central mound in the middle [1]. The inner portion of the enclosure was filled with small to medium sized rocks and boulders, reaching a height of 0.5 meters. The central area was excavated and the burial pit identified, with evidence for animal-caused bioturbation present. Of the two individuals recovered, one was centrally located and in situ, while the other skeleton was more fragmentary. Recovered from the burial pit were ~10 sheep or goat astragali [1]. We collected dental calculus from individuals A (sample ID: Z691 / DA-ALT-004) and B (sample ID: Z690 / DA-ALT-002).

Located nearby was the site of ***Takhilgat Uzuur 5***, which included burials of individuals from multiple time periods. The earliest burial (TU-5, Tomb 31) included a single individual associated with the Early Bronze Age (EBA), with a conventional radiocarbon age of 4133±35 (2872-2619 cal BCE). Tomb 31 was a rectangular-shaped mound with vertical slabs marking the inner and outer enclosures [1]. The interiors of the enclosures were filled with smaller stones, and the outer enclosure measured 7.4 x 6.8 meters. At the top of the burial shaft, sheep bones and ceramic fragments were recovered alongside a human mandible. The remainder of the human remains were recovered below this, intermixed with evidence for carbonized wood. A complete canine (dog or fox) skeleton was also recovered inside the burial [1]. The cultural affiliation of this burial is unclear, but has been discussed as Afanasievo due to the time period and locale. We collected dental calculus from the Tomb 31 individual as sample ID: Z687 / DA-TAK-05.

**Early Bronze Age - Khemceg [Chemurchek] Culture**

Slightly later in time, we analyzed the calculus from two individuals associated with the Khemceg culture and dating to the Early Bronze Age (~2600-2450 cal BC). Individuals were from the sites of Avyn Khukh Uul and Bayan Ondor.

The site of ***Avyn Khukh Uul*** included burials that spanned several time periods and archaeological cultures. Dental calculus from one individual in a Khemceg (Chemurchek) culture burial (AKHU-5, Tomb 2, Level 7) (sample ID: Z696/DA-AKH-020) is from the Early Bronze Age with a radiocarbon date from 2623-2457 cal BCE (3998±35 uncal BP). This burial has a circular stone mound that measures 17 meters in diameter and reaches a height of 1.6 meters [2]. The mount resembles a cone that is missing its top and there is a depression in the center with a depth of one meter. The mound consists of small to medium rock debris, likely collected from the nearby mountain. Under the center of the mound was a burial chamber made of standing slabs. The burial chamber measures 3.4 x 1.8 meters, with a depth of 1.5 meters [2], and contained individuals from several time periods, with the upper levels dating to the Early Iron Age (n=1) and lower levels to the Early Bronze Age (n=3). At the top of the slabs covering the burial, fragmented cattle and horse bones were recovered alongside ceramic sherds and an iron knife. At the lower levels of the burial, animal bones including cattle teeth were recovered alongside the first set of human remains (individual 1). At the lowest level of the burial (level 7) at least three individuals were identified. The remains from individuals 2 and 3 were mixed and fragmentary [2]. Below this level, a final individual (4) was recovered alongside animal remains including cattle and sheep/goat bones. We physically analyzed all four sets of human remains, but dental calculus could only be collected from Individual 4, at the lowest level of the grave.

One individual from the site of ***Bayan Ondor*** was examined (DA-BAY-001), and was associated with the Khemceg (Chemurchek) culture (EBA) based on recovered artifacts and burial style. Confirming this temporal association, the burial was dated to 4011 uncal BP and calibrated to 2630-2463 cal BCE [2]. The surface structure of the burial is complex, with a central mound measuring 20 meters in diameter and a height of 1.4 meters. A peripheral external enclosure of stone circles the main mound at approximately 5 meters distance from the mound center. The central mound consists of soil and stones and at its center is a rectangular structure marked by the upper portion of vertical slabs [2]. The central stone chamber measures 2.5 x 1.3 meters and was covered by stone slabs. Human remains (from Individual 1) were recovered at a depth of 30 cm alongside cattle bones, and below this was Individual 2, who was buried in the NW corner of the grave with a stone bead. Beneath this individual were animal bones and two stone bowls [2]. At the final level a third individual was recovered, with a small flint scraper. We analyzed two dental calculus samples from Individual 1 as sample DA-BAY-01 and laboratory numbers Z692 and DA513.

**Middle Bronze Age - Sagsai culture**

Nine individuals dating to the Middle Bronze Age (~1500-830 BCE) were studied from four different archaeological sites. This includes individuals from Takhilgat Uzuur 5 (n=3), Tsagaan Asga (n=4), Avyn Khukh Uul (n=1) and Khokh Uzuur (n=1).

The site of ***Tsagaan Asga*** included burials from a single period, the Middle Bronze Age Sagsai culture. We analyzed four individuals (TSA 2012 Tomb 12; TSA 2014 Tombs 5, 15, and 17) from the site that dated from ~1505 to 1135 cal BCE. Excavation at the site in 2012 uncovered several burial monuments, including Tomb 12. This monument was square in shape, measuring 4.7 x 5.1 meters and constructed of river cobbles along the edges and center. The internal fill was made of smaller stones and pebbles, with a total height of 0.3 meters. The burial chamber was located in the center, with the body laid in partially prone position. The person is partially on their right side, with the right arm underneath the body and the head facing left. No artifacts were recovered [3]. The tomb produced a conventional radiocarbon age of 3170±30 (1504-1328 cal BCE). Dental calculus was collected from the individual from Tomb 12 under sample number DA-TSA-10 and analyzed twice as laboratory numbers Z682 and DA380.

Excavations in 2014 included Tomb 5, which was a low circular mound with a height of 0.3 meters and a diameter of 13.2 meters [3]. Encompassing the low mound was an outer ring of lighter colored stones, and a smaller ring of stones that measured ~5.4 meters in diameter. Under the smaller ring of stones in the center was the burial chamber covered by stone slabs [3]. The body was in a prone position, with the head on its right side and legs extended. No artifacts were recovered, but some organic materials found under the body included leather and felt [3]. The tomb produced a conventional radiocarbon age of 3030±30 (1399-1134 cal BCE). Dental calculus was collected from the individual from Tomb 5 under sample number DA-TSA-33 and analyzed twice as laboratory numbers Z683 and DA379.

Tomb 15 had surface features including a round cobble covered grave that measures 9.35 meters in diameter and has a height of 0.4 meters. In the center of the grave, under the cobbles, was a layer of flat stones covering the burial shaft [3]. The individual was placed in the burial on their right side with arms extended and legs slightly flexed. Recovered from the burial was a stone disk with a hole in the center that looks like a weight for a loom or a fishing net. A bone point was recovered from between the ribs, suggesting that this individual died from their wound [3]. The tomb produced a conventional radiocarbon age of 3090±30 (1424-1270 cal BCE). Dental calculus was collected from the individual from Tomb 15 under sample number DA-TSA-58 and analyzed as laboratory number Z685.

Also excavated in 2014 was Tomb 17, which was circular and covered in cobbles. The diameter of the cobble-covered surface was 9.9 meters, with a height of 0.3 meters [3]. After excavation of most of the cobbles, there remained a peripheral stone ring, and at the center of the circle, several flat stones covered the burial chamber. The burial chamber was lined with thick stone slabs and boulders. The individual was laid in the burial shaft on their right side with arms and legs slightly flexed [3]. The tomb gave a conventional radiocarbon age of 2920±30 (1214-1015 cal BCE). Dental calculus was collected from the individual from Tomb 17 under sample number DA-TSA-51and analyzed as laboratory number Z684.

At the site of ***Takhilgat Uzuur 5***, there were three burials that date to the Middle Bronze Age (TU-5, Tomb 25; TU-5, Tomb 15; TU-5, Tomb 17). Tomb 15 consisted of a square enclosure with steles in each corner and measured 6 x 6 meters. The surface of the tomb was covered in rock debris and had a maximum height of 0.4 meters [4]. In the center of the tomb was an oval funerary pit that was lined on the surface with stones. The individual was placed on their left side with arms and legs flexed [4]. No artifacts were recovered from this Sagsai culture grave that has a conventional date of 3081±35 (1427-1234 cal BCE). Dental calculus was collected from this individual under laboratory number Z686 and sample number DA-TAK-001.

Tomb 17 gave conventional dates of 2965±35 (1366-1050 cal BCE) and is associated with the Sagsai culture. The tomb consists of a large round stone enclosure that measures 13 meters in diameter, with a semi-circular kurgan in the center [4]. Small slabs cover the area between the enclosure and the central mound. The central mound reaches a height of 0.6 meters and consisted of a circle of slabs around the funeral pit that was also covered with slabs [4]. The individual was placed in a supine position with their right arm across their waist. The body was placed on the right side of the burial pit and a stone was placed on the left side [4] No artifacts were recovered from this tomb. Dental calculus was collected from this individual as sample number DA-TAK-13 and laboratory number 688.

Tomb 25 consists of a circular mound of stones measuring 6.4 meters in diameter, with large boulders marking its outer limits. In the center of the mound was the burial chamber, covered by stone slabs. Beneath the stone slabs, the individual was laid on their left side, with arms and legs flexed. No artifacts were recovered from this Sagsai grave, which gave a conventional date of 3083±35 (1428-1235 cal BCE). Dental calculus was collected as sample number DA-TAK-21 and laboratory number Z689.

The site of ***Avyn Khukh Uul*** is located in Bulgan sum of Khovd province. It included burials that spanned several time periods, and had one burial of a single individual (AKHU-4, Tomb 2, Levels 1-6) dated to the Middle Bronze Age (2938 uncal BP / 1259-1016 cal BCE), which was given sample number DA-AKH-01 and laboratory number Z694.. The surface structure of the tomb was a circular stone mound measuring 8.4m in diameter, with a height of 0.6 meters [2]. Along the edge of the mound there were large boulders, with the internal structure consisting of smaller rocks and pebbles. Once the smaller stones were removed, it was evident that a boulder ring was located on the periphery, with a smaller oval structure in the center [2]. Human remains identified within the central oval structure were only partially in situ. No artifacts were recovered from this burial, but it was identified as belonging to the Middle Bronze Age Bayantümbe culture [2].

The site of ***Khokh Uzuur*** included only a single burial mound from the Sagsai culture, with Khemceg and Xiongnu graves nearby. The burial mound is conical in shape with a diameter of 7.6 meters and a height of 1.3 meters. The mound consists of rocks and sand, with a stone circle underneath the mound that measures 2.8 x 2 meters [2]. The burial chamber was covered by small stone slabs, and within were the remains of an adult individual. The body was laid on its right side with extended legs and arms. No artifacts were recovered from this burial, but it was dated to 2908 uncal BP, with calibrated dates of 1216-1000 cal BCE [2]. We analyzed dental calculus from this individual with sample and laboratory numbers DA-KHO-01 and Z693, respectively.

**Late Iron Age - Xiongnu culture**

The site of ***Avyn Khukh Uul*** included burials from the Middle Bronze Age and later periods [5]. One burial (AKHU-7, Tomb 16, Level 8) contained an individual from the Late Iron Age (LIA) Xiongnu era (49 cal BCE - 121 cal CE; 1985±35 uncal BP). The surface of the grave was small and circular, approximately 3.2 meters in diameter and 0.2 meters in height [2]. The burial shaft was covered with stone slabs, and the fewer slabs remaining at the north end, suggesting looting. The individual was interred in a wooden plank coffin within the shaft pit, which was surrounded by large stone slabs and boulders. The body was laid in a supine position with arms laid out at their side. Within the coffin, a bone tube, iron knife, iron ring, and iron plate were recovered. Just to the north of the coffin were fragments of sheep/goat mandibles from livestock offerings.

***Shombuuzyn-Belchir*** (SBR) is a small cemetery with 33 standard graves of Xiongnu individuals, dating roughly to the later era of the Xiongnu empire (ca. 50 BCE – 200 CE). Male and female individuals spanning all ages were interred, as well as children and infants. Individuals were buried in graves with surface features of either large or small stone rings, and many of the inner chambers included a wooden coffin or a stone cist. Whereas Xiongnu burials generally contain a range of animal offerings, including sheep/goat, cattle, and horses, the graves at SBR yielded only sheep/goat offerings. Although no horse offerings were found at the cemetery, many of the burials contained iron horse-riding gear. We analyzed the calculus of individuals from six tombs (SBR 7, 8, 13, 15, 19 and 26). Only a single tomb had evidence of dietary proteins (SBR 13) and only one has been radiocarbon dated (SBR15) with a range of dates from 133-213 cal CE. Grave 13 (DA-SBR-001, Z697) contained an older male (35-45) with no animal offerings. Broken beams of a wooden cart were set overtop the stone cist, and beside the deceased were laid an iron-headed spear, a bone-reinforced composite bow, and a set of iron and bone arrowheads [6].

**Supporting Note 2. Demographic Change**

Shifts in the population structure in the past are dependent on archaeological data and the primary source of this information for Mongolia comes from burials. There is a documented increase in population, based on burial sites, for the western portion of the country (Fig. 2). This is further supported by a comparison to systematic survey results from another part of the country. Previously published survey data [7] for the Baga Gazaryn Chuluu area of the Middle Gobi, is a region that is ecologically similar to the Altai Mountains in terms of aridity and vegetation. Systematic pedestrian surveys were conducted across an area of over 200 km^2^ and documented several hundred features of the Bronze and Iron Ages [7] (see p.36-42).

Surveys identified no Neolithic or Early Bronze Age sites in the region, yet they documented 83 burials from the Middle Bronze Age Ulaanzukh culture dated between ca. 1400-1050 BCE. Overall, the total number of burial monuments increases significantly over time from the Middle to Late Bronze Age, with 340 Late Bronze Age khirgisuur burials, that date from ca. 1350-800 BCE. The number of Early Iron Age Slab Graves (n=287; 1000-200 BCE) and Late Iron Age Xiongnu graves (n=326; 300 BCE - 100 CE) remain relatively the same. Thus, the most significant increase occurs from the Middle to Late Bronze Ages.

While the highest numbers of burial monuments are Khirgisurs, these are easily identifiable on the surface. However, not every khirgisuur contains human remains. Thus, the high number (~340) might be a slight overestimate of the number of burials relative to earlier and later periods. Nevertheless, even if only half of these monuments contains a burial – the numbers have still increased significantly.

Several survey projects in northern Mongolia have further demonstrated that even in locales where there is a decrease in the number of mortuary sites, from Late Bronze Age to Xiongnu, the number of habitation sites remained roughly the same or increased [8,9]. Intensive habitation surveys and demographic indexing for data in Khanuy Valley of central Mongolia also demonstrates an increase in population form the Late Bronze Age to Xiongnu era [10].


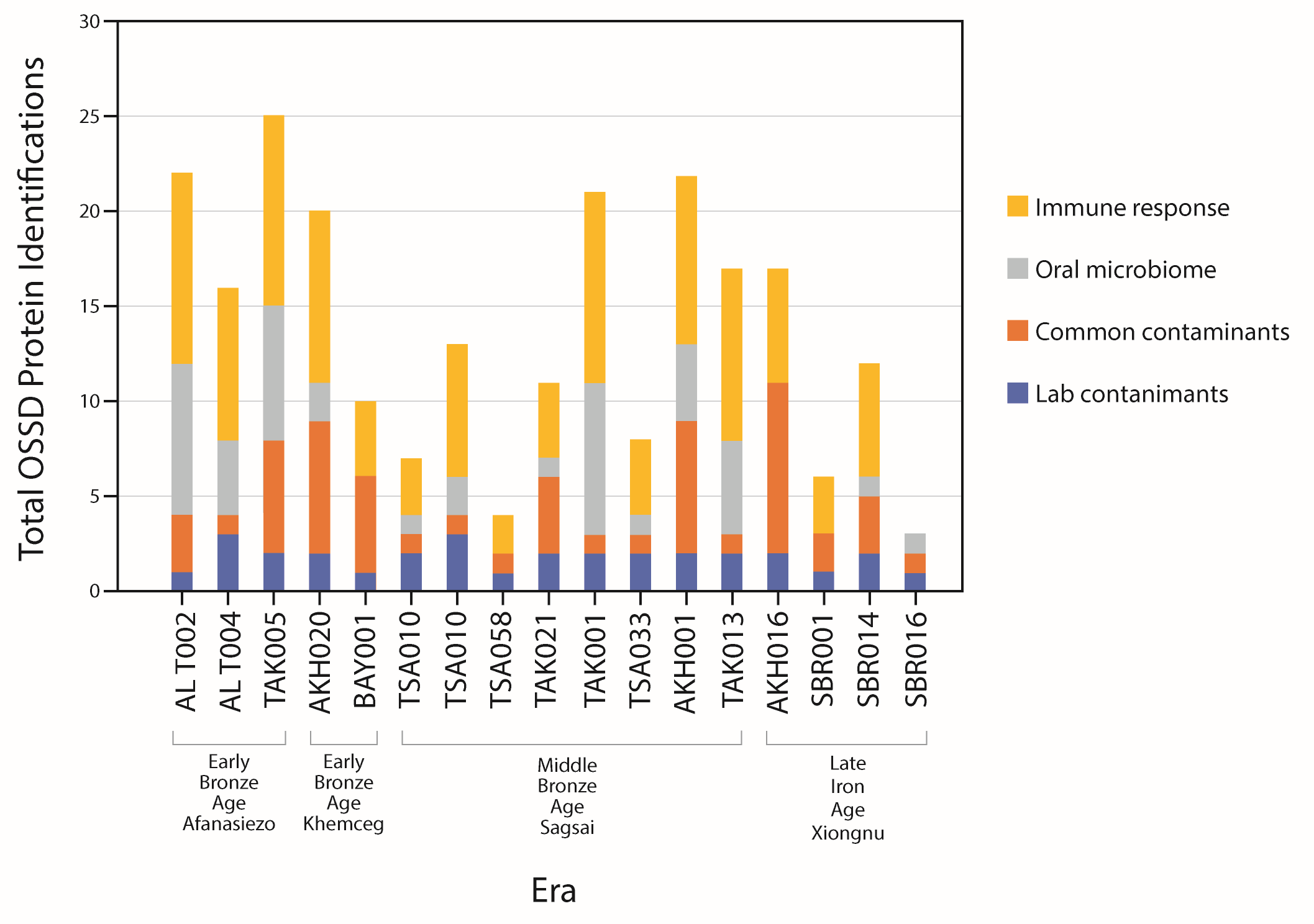


**S1 Fig. Total proteins per sample based on OSSD results**


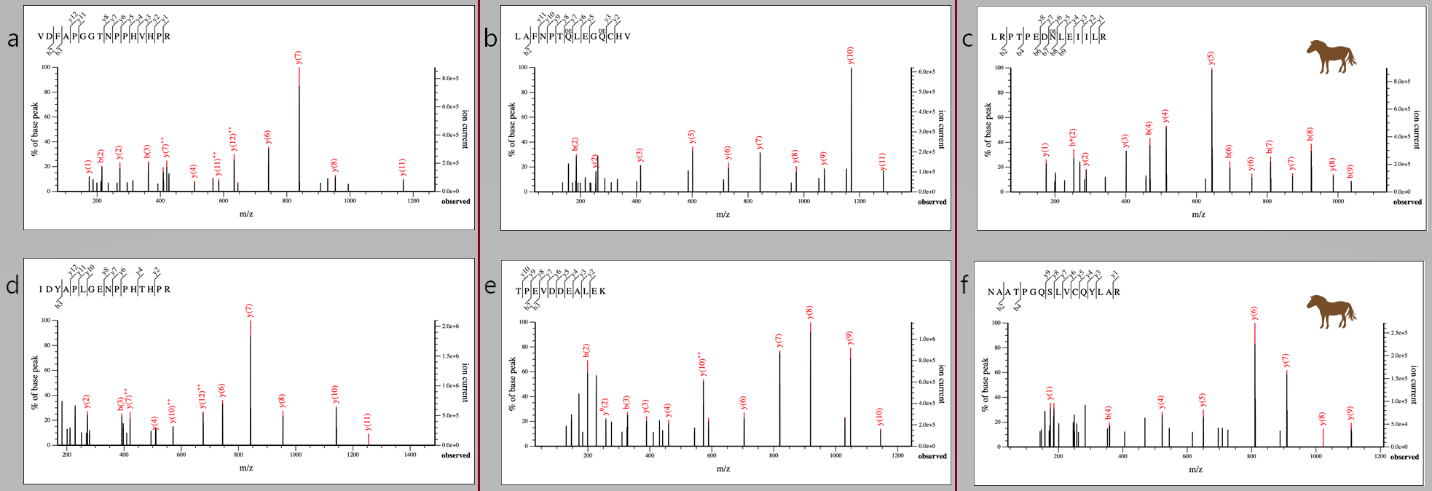


**S2 Fig. Spectra from dietary peptides recovered**

(a,d) DA687 (a) Putative Germin-like protein 3-4 specific to Pocaecae, (d) Germin-like protein 8-10; (b,e) Z691 (b) final tryptic peptide from BLG, specific to Caprinae (*Ovis* or *Capra*), (e) Most commonly recovered BLG peptide, which is specific to Bovinae (cow, yak, water buffalo). Importantly the second Aspartic Acid (D) residue in the sequence has the same mass as a deamidated Aparagine (N) which would be specific to *Ovis*; (c,f) DA379 both spectra are for peptides from BLG I specific to *Equus*. Horses produce both BLG I and II proteins, which are genetically present in ruminants, only the BLG I is expressed.

**Supporting Tables:**

**S1 Table. Results of OSSD with total proteins identified, score (%), and indication of whether the sample passed or failed the initial assessment**

| **Lab ID** | **Sample** | **Dairy** | **Total protein IDS Mascot OSSD*** | **Lab contaminants** | **Common contaminants** | **Oral microbiome** | **Immune response** | **Score (%)** | **PASS / FAIL** |
| --- | --- | --- | --- | --- | --- | --- | --- | --- | --- |
| DA-ALT-02 | Z690 | Yes | 22 | 1 | 3 | 8 | 10 | 82 | PASS |
| DA-ALT-04 | Z691 | Yes | 16 | 3 | 1 | 4 | 8 | 75 | PASS |
| DA-TAK-05 | Z687 | Yes | 25 | 2 | 6 | 7 | 10 | 68 | PASS |
| DA-AKH-20 | Z696 | Yes | 20 | 2 | 7 | 2 | 9 | 55 | PASS |
| DA-BAY-01 | DA513 | No | 11 | 1 | 5 | 0 | 4 | 36 | FAIL |
| DA-BAY-01 | Z692 | Failed Extraction | | | | | | | - |
| DA-TSA-10 | Z682 | No | 7 | 2 | 1 | 1 | 3 | 57 | PASS |
| DA-TSA-10 | DA380 | Yes | 12 | 3 | 1 | 2 | 7 | 75 | PASS |
| DA-TSA-58 | Z685 | Yes | 4 | 1 | 1 | 0 | 2 | 50 | PASS |
| DA-TAK-21 | Z689 | Yes | 11 | 2 | 4 | 1 | 4 | 45 | PASS |
| DA-TAK-01 | Z686 | Yes | 21 | 2 | 1 | 8 | 10 | 86 | PASS |
| DA-TSA-33 | DA379 | Yes | 8 | 2 | 1 | 1 | 4 | 63 | PASS |
| DA-TSA-33 | Z683 | Failed Extraction | | | | | | | - |
| DA-AKH-01 | Z694 | Yes | 22 | 2 | 7 | 4 | 9 | 59 | PASS |
| DA-KHO-01 | Z693 | Failed Extraction | | | | | | | - |
| DA-TSA-51 | Z684 | Failed Extraction | | | | | | | - |
| DA-TAK-13 | Z688 | Yes | 17 | 2 | 1 | 5 | 9 | 82 | PASS |
| DA-AKH-16 | Z695 | No | 17 | 2 | 9 | 0 | 6 | 35 | FAIL |
| DA-SBR-01 | Z697 | Yes | 6 | 1 | 2 | 0 | 3 | 50 | PASS |
| DA-SBR-04 | Z698 | Failed Extraction | | | | | | | - |
| DA-SBR-07 | Z699 | Failed Extraction | | | | | | | - |
| DA-SBR-14 | Z700 | No | 12 | 2 | 3 | 1 | 6 | 58 | PASS |
| DA-SBR-16 | Z701 | No | 3 | 1 | 1 | 1 | 0 | 33 | FAIL |
| DA-SBR-17 | Z702 | Failed Extraction | | | | | | | - |

**S2 Table. Radiocarbon Results**

| **Lab Number** | **Sample Number** | **Original Designation** | **Culture** | **Conventional Age** | **±** | **Radiocarbon Laboratory** | **Calibrated (IntCal20 / Calib 8.2)** |
| --- | --- | --- | --- | --- | --- | --- | --- |
| **Z690** | **DA-ALT-002** | **Altan Tolgoi-2, AT-2, Person B** | **Afanasievo (EBA)** | − | **-** | **Individual associated with DA-ALT-04** | **2912-2671 cal BC 95.4 (2 sigma)** |
| **Z691** | **DA-ALT-004** | **Altan Tolgoi-2, AT-2, Person A** | **Afanasievo (EBA)** | **4228** | **40** | **COL1531.1.1** | **2912-2671 cal BC 95.4 (2 sigma)** |
| **Z687** | **DA-TAK-005** | **Takhilgat Uzuur-5, TU-5, Tomb 31** | **(EBA)** | **4133** | **35** | **COL1538.1.1** | **2873-2580 cal BC 95.4 (2 sigma)** |
| **Z696** | **DA-AKH-020** | **Avyn Khukh Uul, AKHU-5, Tomb 2, level 7** | **Khemceg / Chemurchek (EBA)** | **3998** | **35** | **COL 2049.1.1** | **2623-2457cal BC 95.4 (2 sigma)** |
|  |  |  |  |  |  |  |  |
| **DA513** | **DA-BAY-001** | **Bayan Ondor** | **Khemceg / Chemurchek (EBA)** | **−** | **−** | **−** | **−** |
| **Z692** | **DA-BAY-001** | **Bayan Ondor** | **Khemceg / Chemurchek (EBA)** | **−** | **−** | **−** | **−** |
| **Z682** | **DA-TSA-10** | **Tsagaan Asga 2012, Tomb 12** | **Sagsai (MBA)** | **3170** | **30** | **Beta-407902** | **1504-1328 cal BC 95.4 (2 sigma)** |
| **DA380** | **DA-TSA-10** | **Tsagaan Asga 2012, Tomb 12** | **Sagsai (MBA)** | **3170** | **30** | **Beta-407902** | **1504-1328 cal BC 95.4 (2 sigma)** |
| **Z685** | **DA-TSA-058** | **Tsagaan Asga 2014, Tomb 15** | **Sagsai (MBA)** | **3090** | **30** | **Beta-407905** | **1424-1270 cal BC 95.4 (2 sigma)** |
| **Z689** | **DA-TAK-021** | **Takhilgat Uzuur-5, TU-5, tomb 25** | **Sagsai (MBA)** | **3083** | **35** | **Cologne COL1536.1.1** | **1428-1235 cal BC 95.4 (2 sigma)** |
| **Z686** | **DA-TAK-001** | **Takhilgat Uzuur-5, TU-5, tomb 15, level 2** | **Sagsai (MBA)** | **3081** | **35** | **Cologne COL1533.1.1** | **1427-1234 cal BC 95.4 (2 sigma)** |
|  |  |  |  |  |  |  |  |
|  |  |  |  |  |  |  |  |
|  |  |  |  |  |  |  |  |
| **Z683** | **DA-TSA-033** | **Tsagaan Asga 2014, Tomb 5, level 4** | **Sagsai (MBA)** | **3030** | **30** | **Beta-407895** | **1399-1134 cal BCE 95.4 (2 sigma)** |
| **DA379** | **DA-TSA-033** | **Tsagaan Asga 2014, Tomb 5, level 4** | **Sagsai (MBA)** | **3030** | **30** | **Beta-407895** | **1399-1134 cal BCE 95.4 (2 sigma)** |
| **Z688** | **DA-TAK-013** | **Takhilgat Uzuur-5, TU-5, tomb 17** | **Sagsai (MBA)** | **2965** | **35** | **COL1534.1.1** | **1366-1050 cal BCE 95.4 (2 sigma)** |
|  |  |  |  |  |  |  |  |
|  |  |  |  |  |  |  |  |
| **Z694** | **DA-AKH-001** | **Avyn Khukh Uul, AKHU-4, tomb 2, levels 1-6** | **Sagsai (MBA)** | **2938** | **37** | **COL2038.1.1** | **1259-1016 cal BCE 95.4 (2 sigma)** |
|  |  |  |  |  |  |  |  |
|  |  |  |  |  |  |  |  |
| **Z684** | **DA-TSA-051** | **Tsagaan Asga 2014, Tomb 17** | **Sagsai (MBA)** | **2920** | **30** | **Beta-407907** | **1214-1015 cal BCE 95.4 (2 sigma)** |
| **Z693** | **DA-KHO-001** | **Khokh Uzuur-1** | **Sagsai (MBA)** | **−** | **−** | **−** | **−** |
| **Z695** | **DA-AKH-016** | **Avyn Khukh Uul, AKHU-7, tomb 16, level 8** | **Xiongnu (LIA)** | **1985** | **35** | **COL 2037.1.1** | **46 cal BCE - 121 cal CE 95.4 (2 sigma)** |
| **Z697** | **DA-SBR-001** | **Shombuuzin-Belchir SBR 13** | **Xiongnu (LIA)** | **−** | **−** | **−** | **−** |
| **Z698** | **DA-SBR-004** | **Shombuuzin-Belchir SBR 7 (2010)** | **Xiongnu (LIA)** | **−** | **−** | **−** | **−** |
| **Z699** | **DA-SBR-007** | **Shombuuzin-Belchir SBR 8 (2010)** | **Xiongnu (LIA)** | **−** | **−** | **−** | **−** |
| **Z700** | **DA-SBR-014** | **Shombuuzin-Belchir SBR 15 (2008)** | **Xiongnu (LIA)** | **1840** | **20** | **Brosseder et al. 2011** | **129 - 243 cal CE 95.4 (2 sigma)** |
| **Z701** | **DA-SBR-016** | **Shombuuzin-Belchir SBR 19 (2008)** | **Xiongnu (LIA)** | **−** | **−** | **−** | **−** |
| **Z702** | **DA-SBR-017** | **Shombuuzin-Belchir SBR 26 (2010)** | **Xiongnu (LIA)** | **−** | **−** | **−** | **−** |

**References**

1. Turbat T, Batsukh D, Bayarkhuu N. Mongolia-European Union joint ‘Eurasiat’ Project Archaeology Field Analysis 2011 Work Report. Ulaanbaatar: Institute of Archaeology of the MAS International Eurasiat Center; 2011.

2. Turbat T. Report to the Gerda Henkel Foundation: Results of the “Aeneolithic and Bronze Age of the Mongolian Altai” project. 2012.

3. Turbat T, Batsukh D, Bayarkhuu N, Enkhbayar G. Mongolia-France joint ‘Eurasiat’ Bronze Grave Project Archaeological Field Analysis Class 2014 Work Report. Ulaanbaatar: Institute of Archaeology of the MAS International Eurasiat Center; 2014.

4. Turbat T, Bemmann J. Report to the Gerda Henkel Foundation: Results of the “Bronze Age of Mongolian Altai” project. 2011.

5. Turbat T, Batsukh D, Bemmann J. Xiongnu burials at Avyn Khökh Uul, Southern Slopes of Mongolian Altai. Arkhaeologiin Sudlal. 2015;35: 295–323.

6. Bayarsaikhan J, Miller BK, Egiimaa T, Konovalov PB, Johannesson E, Machicek M, et al. Xiongnu monuments at Shombuuzyn Belchir. 11: 156-183. Nüüdelchdiin öv sudlal. 2011;11: 156–183.

7. Amartuvshin C, Honeychurch W. Survey and Bioarchaeology in the Middle Gobi: The Baga Gazaryn Chuluu Project. XXVII. Ulaanbaatar: Studia Archaeologica; 2010.

8. Clark JK. Modeling late prehistoric and early historic pastoral adaptations in northern Mongolia’s Darkhad Depression. Ph.D., University of Pittsburgh. Available: http://www.proquest.com/docview/1666829007/abstract/90B8533470E84C11PQ/1

9. Gardner WRM. Early political complexity and community organization on the Mongolian steppe. Yale University; 2016.

10. Houle J-L, Broderick LG. Settlement patterns and domestic economy of the Xiongnu in Khanui Valley, Mongolia. In: Miller BK, Brosseder U, editors. Xiongnu Archaeology: Multidisciplinary Perspectives of the First Steppe Empire. Bonn: VFG-Arch Press; 2011. pp. 137–152.
